# A Knowledge, Attitude, and Practice Study on Trauma-Informed Care Among Nurses Working in a Hospital in Lucknow District

**DOI:** 10.7759/cureus.84652

**Published:** 2025-05-22

**Authors:** Rajeev Misra, Akanksha Mishra, Rajgopal Reddy, Divyanshi Singh

**Affiliations:** 1 Community Medicine and Public Health, King George's Medical University, Lucknow, IND; 2 Nutrition, Isabella Thoburn College, Lucknow, IND; 3 Clinical Operations, Chandan Hospital, Lucknow, IND; 4 Psychology, National Post Graduate College, Lucknow, IND

**Keywords:** knowledge, nurses, post-traumatic stress disorder, re-traumatization, trauma-informed care

## Abstract

Background

Trauma-informed care (TIC) is a framework that acknowledges the widespread impact of trauma and integrates knowledge about its effects into healthcare practices. As frontline caregivers, nurses frequently encounter patients with trauma histories. Their knowledge, attitude, and practice (KAP) regarding TIC are crucial in ensuring compassionate, effective, and patient-centered care. However, there is limited research assessing the understanding and implementation of TIC among nurses in Indian healthcare settings, particularly in Lucknow District.

Materials and methods

A cross-sectional, web-based survey was conducted from March 2024 to July 2024, using a random sampling method to assess nurses’ KAP regarding TIC at Chandan and Fatima Hospitals.

Results

Among 208 nurses, 52.88% had 1-5 years of experience. No significant association was found between age and trauma-related perceptions (*p*> 0.05). Education (*p* = 0.049) and gender (*p* = 0.004) significantly influenced TIC techniques, with general nursing and midwifery (GNM) nurses and females predominantly using a broader range of therapeutic approaches.

Conclusions

This study is significant because it will provide evidence-based insights into the preparedness of nurses in Lucknow District regarding TIC. The findings can contribute to improved nursing education, hospital policies, and patient outcomes by promoting a more trauma-sensitive healthcare environment.

## Introduction

Trauma occurs when an event, sequence of events, or set of circumstances affects a person’s functioning and mental, physical, emotional, or spiritual well-being [1]. Increased behavioral and psychological symptoms in teenagers are indicative of repeated trauma exposure [2]. For patients and their families, medical procedures can be traumatizing [3].

The strength-based approach known as trauma-informed care (TIC) is based on an understanding of responsiveness to the trauma impact. It is focused on physical, psychological, and emotional safety for both providers and survivors, and that creates opportunities for survivors to rebuild a sense of control and empowerment [4].

TIC reveals the frequency and effects of traumatic experiences in clinical practice. It emphasizes giving patients a sense of security, control, and autonomy over their lives and medical decisions [5]. A trauma-informed approach to nursing care consists of trauma-specific interventions, which include assessment, treatment, and recovery support. It introduces key trauma principles of the targeted organizational culture [6]. According to Substance Abuse and Mental Health Services Administration (SAMHSA), the goals of TIC are to: a) reduce retraumatization, (b) highlight survivor strengths and resilience, (c) promote healing and recovery, and (d) support the development of healthy short- and long-term coping mechanisms [6].

SAMHSA created the framework that outlines the six main TIC principles: (a) safety; (b) openness and trust; (c) peer support; (d) cooperation and reciprocity; (e) autonomy, voice, and decision-making; and (f) issues related to culture, gender, and history [6]. Transparency and trust are essential, and clinicians should speak candidly and early on with teenagers regarding their differential diagnoses and final diagnosis [7]. Many medical professionals might feel unprepared to deal with patients’ past trauma and the risk of re-traumatization [8].

A review of the literature revealed one qualitative study that evaluated nursing knowledge and experience regarding TIC [9]. Nurses have numerous opportunities to impact patients’ and colleagues’ experiences, so nursing is a crucial field for implementing a trauma-informed approach [10].

According to some reports, trauma can occur at any point in a person’s life [11,12]. An individual’s psychological and physiological development may be altered by early traumatic experiences, which can lead to increased risk behaviors and a host of negative emotional, social, economic, and health outcomes. According to Harris and Fallot (2001), nurses are positioned to play a significant role in advancing TIC within healthcare services because they are direct care providers who can work from a holistic perspective. Regretfully, some research indicates that nurses frequently struggle to understand and apply TIC concepts in their daily work because of ambiguous definitions [13].

Implementing the TIC framework can build nurses’ knowledge about past trauma and the impact of trauma on a patient’s ongoing mental illness. The main aim of TIC is to avoid re-traumatizing the patient through their episode of care. A TIC education package can give nurses content describing the interplay of neurological, biological, psychological, and social effects of trauma that may decrease the likelihood of re-traumatization [14]. Trauma is a widespread, yet often unrecognized, public health issue that significantly impacts patient outcomes and the quality of healthcare delivery. Nurses, as frontline healthcare providers, play a crucial role in identifying and responding to trauma-related symptoms. However, despite the growing awareness of trauma-informed practices, a significant gap persists in the understanding, perception, and implementation of TIC among nursing professionals, especially in resource-constrained and high-stress environments. Ultimately, the findings will support the integration of trauma-informed principles into routine nursing care, fostering safer and more supportive healthcare environments for both patients and providers.

Objectives

The primary objectives are to assess the level of knowledge related to trauma-informed care among nurses, evaluate the attitudes of nurses toward trauma-informed care practices, and assess the practices of nurses regarding the implementation of trauma-informed care in clinical settings.

The secondary objective is to explore the trauma-related awareness of trauma-informed care.

## Materials and methods

Study design

A cross-sectional, web-based survey was conducted to assess the level of knowledge, attitude, and practice (KAP) regarding TIC among nurses. The survey was conducted between March 2024 and July 2024. A link to a Google Form survey was distributed across social networking platforms and WhatsApp groups, and the data was summarized using frequency, percentage, and chi-square tests.

The study population included nurses, and the study area was the Chandan and Fatima Hospitals. The duration of the study was from March 2024 to July 2024

The sample size of 208 was calculated using the formula:

$N = \frac{z^2 p(1-p)}{E^2(N-1) + z^2 p(1-p)}$

where N (population size) = 450, estimated proportion = 0.5, Z = confidence level = 95% = 1.96, E = margin of error = 0.05

The sample size was small because the study was completed within a short time frame. The sampling population was nurses who were working in hospitals. The sample was selected using a random sampling method to ensure unbiased representation. Specifically, the lottery method was employed, wherein the names of all eligible hospitals in the target area were written on identical slips of paper. These slips were then placed in a container and thoroughly mixed to eliminate any selection bias. Two slips were randomly selected to choose the participating hospitals. The hospitals selected through this process were Chandan Hospital and Fatima Hospital. This process ensured the selection was entirely random and free from any predetermined preferences or influences.

Tool used

Data were collected using a valid and reliable questionnaire (Appendices). The questionnaire contained open-ended, closed-ended, and multiple-choice questions with predefined answers that revealed information about the KAP of TIC among nurses. The questionnaire used in our study was adapted from existing, previously published literature on trauma-informed care and KAP frameworks.

Questionnaire design based on knowledge, attitude, and perception criteria 

Knowledge was assessed in terms of understanding of TIC core principles (e.g., safety, trust, peer support, empowerment, cultural sensitivity). Attitude was evaluated by gauging the participants’ openness, perceived relevance, and belief in the effectiveness of TIC in clinical practice. Perception included awareness of trauma's impact on patient care and the need for trauma-informed approaches in nursing.

Sampling method

The sampling method used was purposive sampling, focusing on nurses who met the criteria of KAP.

Inclusion and exclusion criteria

The study included nurses from Chandan and Fatima Hospitals who were willing to provide informed consent. Participants of all age groups were considered eligible, provided they had no major physical or psychiatric illnesses that might interfere with their ability to participate effectively. Nurses who declined to give consent, those who were diagnosed with significant physical or psychiatric conditions, or those who were employed at hospitals other than Chandan and Fatima were excluded from the study.

Data analysis

The collected data were systematically compiled, coded, and entered into Microsoft Excel (Microsoft Corporation, Redmond, WA, US) for later analysis using the Statistical Package for the Social Sciences (SPSS), Version 26 (IBM Corp., Armonk, NY. Descriptive statistics such as frequency, percentage, mean, and standard deviation were used to summarize the demographic characteristics and key variables related to KAP concerning TIC.

Inferential statistics were then applied to determine associations between demographic variables (such as age, education, experience, and gender) and responses related to TIC. The chi-square test was employed to assess the statistical significance of these associations. A p-value of less than 0.05 was considered statistically significant. The findings were interpreted to identify trends, gaps in knowledge, and variations in the practice of TIC among different subgroups of nurses.

Research question

What are the levels of knowledge, attitude, and practice regarding trauma-informed care among nurses working in hospitals in Lucknow District and what factors influence their adoption of trauma-informed approaches in patient care?

## Results

This study explored nurses’ perceptions, knowledge, attitudes, and practices related to TIC. The analysis focused on identifying key elements recognized by the nurses, their practical approaches, and how factors such as gender, experience, and education level influenced the use of TIC techniques.

Work experience of participants

Table 1 outlines the professional experience of the participating nurses. A majority (52.88%) had between 1 to 5 years of experience, followed by 33.17% with less than 1 year. Very few had over 10 years of experience, indicating that the workforce primarily comprised early-career professionals.

**Table 1:** Distribution of participants by work experience

Experience	Frequency	Percentage
Less than 1 year	69	33.17%
1–5 years	110	52.88%
10–15 years	28	13.46%
More than 15 years	1	0.48%
Total	208	100%

Association between age and trauma-related perceptions

Table 2 illustrates the association between participants' age and their perceptions regarding trauma and trauma-informed care. The chi-square test of independence revealed no statistically significant associations (p > 0.05) across all items, suggesting that trauma-related perceptions were generally consistent across different age groups.

**Table 2:** Association between age and trauma-related perceptions (test used: chi-square test of independence) TIC: trauma-informed care

Question	χ²	df	p-value
Do you think exposure to trauma is common?	15.12	16	0.516
Do you think trauma can affect physical well-being?	16.56	16	0.414
Do you think trauma can affect mental well-being?	12.69	16	0.696
Do you think trauma can affect emotional well-being?	7.30	16	0.967
Do you think trauma can have lifelong effects across generations?	15.20	16	0.510
Do you assume patients who are alone have trauma history?	13.84	16	0.611
Is trauma presentation influenced by age, gender, or culture?	14.40	16	0.569
Is mental health (depression, stress, etc.) linked to trauma?	14.07	16	0.593
Can re-traumatization occur unintentionally?	14.40	16	0.569
Is recovery from trauma possible?	11.47	16	0.780
Are patients experts in their healing?	14.17	16	0.586
Are paths to recovery different for everyone?	13.61	16	0.628
Is informed choice essential in TIC?	13.30	16	0.651
Should nurses know specific trauma history?	21.81	16	0.149
Does TIC involve understanding patient and provider safety?	16.46	16	0.422
Should TIC create healing environments?	11.82	16	0.756
What techniques do you use while giving TIC?	9.74	20	0.973
Do you maintain transparency with patients?	16.81	16	0.398
Do you offer choices and respect decisions?	12.61	16	0.702
Do you inform patients before all actions?	11.49	16	0.778

Association between educational qualification and TIC techniques

As shown in Table 3, nurses with different educational backgrounds used varying techniques while practicing trauma-informed care (TIC). The majority of general nursing and midwifery (GNM)-qualified nurses reported using multiple techniques such as deep breathing and relaxation. A chi-square test revealed a statistically significant association between education level and TIC techniques (χ² = 37.74, df = 25, p = 0.049), indicating that educational background influenced the types of techniques employed.

**Table 3:** Association between education level and techniques used for trauma-informed care (test used: chi-square test of independence) ANM: Auxiliary Nurse Midwife; GNM: General Nursing and Midwifery

Education Level	All Techniques	Muscle Relaxation	Deep Breathing	Meditation	Diversion Therapy/Music	None	Total
GNM	118	5	7	2	0	1	133
Other	4	2	3	0	0	0	9
BSc Nursing	27	3	4	2	1	0	37
Diploma in Nursing	6	2	4	0	0	0	12
MSc Nursing	3	0	0	0	0	0	3
ANM	11	0	2	1	0	0	14
Total	169	12	20	5	1	1	208

Association between gender and TIC techniques

Table 4 represents the association between gender and the techniques used in trauma-informed care. Female nurses were more likely to use multiple techniques ("all the above") than male nurses. The chi-square analysis showed a statistically significant association (χ² = 17.19, df = 5, p = 0.004), indicating that gender had an impact on the choice of TIC techniques.

**Table 4:** Association between gender and techniques used for trauma-informed care (test used: chi-square test of independence)

Gender	All Techniques	Muscle Relaxation	Deep Breathing	Meditation	Diversion Therapy/Music	None	Total
Female	146	6	18	4	0	1	175
Male	23	6	2	1	1	0	33
Total	169	12	20	5	1	1	208

## Discussion

This study investigated nurses’ perceptions and experiences with TIC. Although most participants were unfamiliar with the formal definition of TIC as described in the literature [15], when asked to explain what they understood, they identified several key elements. These included the importance of understanding trauma in practice, recognizing trauma symptoms, building rapport, adapting care approaches, and preventing re-traumatization [16].

The study sample was predominantly composed of young nurses with less experience, which may have influenced their practices and understanding of TIC. Also, the results demonstrated how significant education is because higher education levels greatly influenced the choice and use of TIC techniques. This shows how important it is to have customized training programs to ensure that TIC is delivered consistently.

The KAP of nursing interruptions related to clinical management levels among nurses indicated significant variations based on socioeconomic factors, experiences in clinical units, working conditions, and training backgrounds [17,18]. Therefore, it is crucial to consider these variables when developing strategies for preventing and managing nursing interruptions among clinical nurses. The total KAP score in this study was highest among the following groups of nurses: those aged 41-45, those with 16-20 years of service, those who do not work night shifts, those who received standardized nursing interruption training, and those who had their leaders’ attention. This also applied to formally employed nurses, chief nurses, nursing managers, and nurses with a first degree of a master’s or higher. In contrast, the lowest KAP score was observed among nurses aged 26-30 years who had 6-10 years of service, worked night shifts, had not received standardized nursing interruption training, and those who strongly disagreed with the item regarding their leaders’ attention, as well as temporary nurses and those holding senior and general positions with a first-degree undergraduate qualification. Therefore, nursing managers should focus more on nurses with low KAP scores related to nursing interruptions.

Gaining insight into nurses’ knowledge and beliefs regarding nursing interruptions can aid in designing effective educational interventions. Moreover, the main factors that influenced nurses’ involvement in continuing education were employer support, financial incentives, and chances for advancement [19]. Thus, to foster enthusiasm among temporary nurses, it is necessary to protect their rights and interests and implement support and reward programs for academic accomplishments.

Gender differences also emerged as significant, pointing to the need for inclusive training strategies to address these variations in practice. Interestingly, despite demographic differences, nurses demonstrated consistent perceptions of trauma-related issues, indicating a shared baseline understanding across the group. The quality and efficacy of patient care, as well as patient safety, are directly impacted by the competency of nurses [20].

This study has several limitations. The sample predominantly included young, less experienced nurses, which may limit the generalizability of findings to more seasoned professionals. Additionally, data were collected from a single geographic location, potentially affecting the applicability of results across different cultural or institutional settings. The absence of a longitudinal design restricts insights into the long-term impact of TIC training. Furthermore, external factors, such as workload, organizational support, or policy frameworks influencing TIC implementation, were not explored. These constraints highlight the need for broader, more diverse research to fully understand the effectiveness and adoption of TIC in varied healthcare contexts.

## Conclusions

This study offers important insights into nurses’ KAP regarding TIC. Despite a limited formal understanding of TIC, most participants recognized its core principles, including identifying trauma symptoms and preventing re-traumatization. The predominance of young nurses with limited experience suggests the need for enhanced training to ensure consistent TIC application. Education level and gender significantly influenced the techniques adopted, indicating the value of customized and inclusive training. Overall, the findings underscore the necessity of integrating TIC into nursing education and ongoing professional development to promote compassionate, trauma-sensitive healthcare across diverse settings. Based on the findings, several recommendations can be made to improve the implementation and understanding of TIC. First, training programs should be tailored to address the specific needs of nurses with varying educational backgrounds, ensuring a standardized approach to TIC techniques such as deep breathing, meditation, and muscle relaxation. Gender-sensitive training should also be incorporated to bridge differences in practice and promote inclusivity. Institutions should consider integrating TIC principles into regular nursing curricula and offering refresher courses to enhance understanding and application across experience levels.

## Appendices

Questionnaire

1.     Name:

2.     Age:

3.     Category:

·       General

·       OBC

·       SC

·       ST

4.     Religion:

·       Hindu

·       Muslim

·       Christian

·       Buddhism

5. Education:

·       GNM

·       ANM

·       Bsc

·       Msc

·       Diploma in nursing

·       Other

6. Job Status:

7. Experience:

·       Less than 1 year

·       1-5 years

·       10-15 years

·       More than 15 years

8. Do you think exposure to trauma is common?

·       Agree

·       Disagree

·       Strongly agree

·       Strongly disagree

·       Neutral

9. Do you think trauma can affect physical well-being?

·       Agree

·       Disagree

·       Strongly agree

·       Strongly disagree

·       Neutral

10. Do you think trauma can affect mental well-being?

·       Agree

·       Disagree

·       Strongly agree

·       Strongly disagree

·       Neutral

11. Do you think trauma can affect emotional well-being?

·       Agree

·       Disagree

·       Strongly agree

·       Strongly disagree

·       Neutral

12. Do you think trauma can have lifelong effects that may span generations?

·       Agree

·       Disagree

·       Strongly agree

·       Strongly disagree

·       Neutral

13. Do you think that it is reasonable to assume that patients who are alone have been exposed to trauma?

·       Agree

·       Disagree

·       Strongly agree

·       Strongly disagree

·       Neutral

14.  Do you think traumatic stress may be present differently in patients depending on age, gender, or culture?

·       Agree

·       Disagree

·       Strongly agree

·       Strongly disagree

·       Neutral

15. Do you think there is any connection between mental health(depression, fear, anxiety, stress) issues and past traumatic experiences?

·       Agree

·       Disagree

·       Strongly agree

·       Strongly disagree

·       Neutral

16.  Re-traumatisation can occur unintentionally.

·       Agree

·       Disagree

·       Strongly agree

·       Strongly disagree

·       Neutral

17. According to you, recovery from trauma is possible.

·       Agree

·       Disagree

·       Strongly agree

·       Strongly disagree

·       Neutral

18. Do you think patients are experts in their own healing/recovery from trauma?

·       Agree

·       Disagree

·       Strongly agree

·       Strongly disagree

·       Neutral

19. Do you think paths to healing/recovery from trauma are different for everyone?

·       Agree

·       Disagree

·       Strongly agree

·       Strongly disagree

·       Neutral

20. Do you think informed choice against the trauma care is essential to healing/recovery from trauma? 

·       Agree

·       Disagree

·       Strongly agree

·       Strongly disagree

·       Neutral

21. Do you think that when using trauma-informed care (TIC), nurses must know specific details of a patient's history of trauma? 

·       Agree

·       Disagree

·       Strongly agree

·       Strongly disagree

·       Neutral

 22. Do you think trauma-informed care (TIC) includes an understanding of the physical, psychological, and emotional safety of both the patient and the provider?

·       Agree

·       Disagree

·       Strongly agree

·       Strongly disagree

·       Neutral

23. Do you think trauma-informed care (TIC) requires providers to recognize, understand, and respond to the effects of trauma? 

·       Agree

·       Disagree

·       Strongly agree

·       Strongly disagree

·       Neutral

24. Do you think trauma-informed care (TIC) aims to create safe environments that promote healing and recovery from trauma exposure? 

·       Agree

·       Disagree

·       Strongly agree

·       Strongly disagree

·       Neutral

25. What are the different techniques you follow while giving trauma-informed care (TIC)?

·       Deep breathing

·       Muscle relaxation

·       Meditation

·       All of the above

·       Other

26. What are the different types of positive resources you give to a traumatically injured patient?

·       Resilient

·       Hobbies

·       Strong character traits

·       All of the above

·       Other

27. Do you maintain transparency in all interactions with patients?

·       Never

·       Seldom

·       Sometimes

·       Often

·       Always

28. Do you offer patient choices and respect their decision?

·       Never

·       Seldom

·       Sometimes

·       Oten

·       Always

29. Do you inform all the patients before performing trauma-informed care (TIC)?

·       Never

·       Seldom

·       Sometimes

·       Often

·       Always
